# Tensor Decomposition-Based Unsupervised Feature Extraction Applied to Single-Cell Gene Expression Analysis

**DOI:** 10.3389/fgene.2019.00864

**Published:** 2019-09-19

**Authors:** Y-h. Taguchi, Turki Turki

**Affiliations:** ^1^Department of Physics, Chuo University, Tokyo, Japan; ^2^Department of Computer Science, King Abdulaziz University, Jeddah, Saudi Arabia

**Keywords:** tensor decomposition, enrichment analysis, single-cell RNA-sequencing, midbrain development, inter-species analysis

## Abstract

Although single-cell RNA sequencing (scRNA-seq) technology is newly invented and a promising one, but because of lack of enough information that labels individual cells, it is hard to interpret the obtained gene expression of each cell. Because of insufficient information available, unsupervised clustering, for example, *t*-distributed stochastic neighbor embedding and uniform manifold approximation and projection, is usually employed to obtain low-dimensional embedding that can help to understand cell–cell relationship. One possible drawback of this strategy is that the outcome is highly dependent upon genes selected for the usage of clustering. In order to fulfill this requirement, there are many methods that performed unsupervised gene selection. In this study, a tensor decomposition (TD)-based unsupervised feature extraction (FE) was applied to the integration of two scRNA-seq expression profiles that measure human and mouse midbrain development. TD-based unsupervised FE could select not only coincident genes between human and mouse but also biologically reliable genes. Coincidence between two species as well as biological reliability of selected genes is increased compared with that using principal component analysis (PCA)-based FE applied to the same data set in the previous study. Since PCA-based unsupervised FE outperformed the other three popular unsupervised gene selection methods, highly variable genes, bimodal genes, and dpFeature, TD-based unsupervised FE can do so as well. In addition to this, 10 transcription factors (TFs) that might regulate selected genes and might contribute to midbrain development were identified. These 10 TFs, BHLHE40, EGR1, GABPA, IRF3, PPARG, REST, RFX5, STAT3, TCF7L2, and ZBTB33, were previously reported to be related to brain functions and diseases. TD-based unsupervised FE is a promising method to integrate two scRNA-seq profiles effectively.

## Introduction

Single-cell RNA sequencing (scRNA-seq) (Sasagawa et al., 2019) is a newly invented technology that enables us to measure the amount of RNA in a single-cell basis. In spite of its promising potential, it is not easy to interpret the measurements. The primary reason of this difficulty is the lack of sufficient information that characterizes individual cells. In contrast to the huge number of cells measured, which is often as many as several thousands, the number of labeling is limited, for example, measurement of conditions as well as the amount of expression of key genes measured by fluorescence-activated cell sorting, whose number is typically as little as tens. This prevents us from selecting genes that characterize the individual cell properties.

In order to deal with samples without suitable numbers of labeling, unsupervised method is frequently used, since it does not make use of labeling information directly. *K*-means clustering and hierarchical clustering are popular methodologies that are often applied to gene expression analysis. The popular clustering methods specifically applied to scRNA-seq are *t*-distributed stochastic neighbor embedding (tSNE) (van der Maaten and Hinton, 2008) and uniform manifold approximation and projection (UMAP) (McInnes et al., 2018), which are known to be useful to get low-dimensional embedding of a set of cells. In spite of that, the obtained clusters are highly dependent upon genes used for clustering. Thus, the next issue is, without labeling (i.e., pre-knowledge), to select genes that might be biologically meaningful.

The various unsupervised gene selection methods applicable to scRNA-seq were invented, for example, highly variable genes, bimodal genes, dpFeature, and principal component analysis (PCA)-based unsupervised feature extraction (FE) (Murakami et al., 2012; Taguchi and Okamoto, 2012; Taguchi and Murakami, 2013; Ishida et al., 2014; Kinoshita et al., 2014; Murakami et al., 2014; Taguchi, 2014; Taguchi and Murakami, 2014; Umeyama et al., 2014; Murakami et al., 2015; Taguchi, 2015; Taguchi et al., 2015a; Taguchi et al., 2015b; Taguchi et al., 2015c; Taguchi et al., 2016; Taguchi, 2016a; Taguchi, 2016b; Taguchi, 2016c; Taguchi, 2016d; Taguchi and Wang, 2017; Taguchi et al., 2017; Taguchi, 2017d; Taguchi and Wang, 2018a; Taguchi, 2018a; Taguchi and Wang, 2018b). Chen et al. (2018) recently compared genes selected by these methods and concluded that the genes selected are very diverse and have their own (unique) biological features. In this sense, it is required to invent more advanced unsupervised gene selection methods that can select more biologically relevant genes.

In this paper, we propose the application of tensor decomposition (TD)-based unsupervised FE (Taguchi, 2017a; Taguchi, 2017b; Taguchi, 2017c; Taguchi, 2017e; Taguchi, 2017f; Taguchi and Ng, 2018; Taguchi, 2018b; Taguchi, 2018c; Taguchi, 2019a). It is an advanced method of PCA-based unsupervised FE for scRNA-seq analysis. For more details about PCA-based unsupervised FE and TD-based unsupervised FE, see the recently published book (Taguchi, 2019b). Especially focusing on the integration of two scRNA-seq profiles, the advantages of TD-based unsupervised FE when compared with PCA-based unsupervised FE are as follows: The former can integrate more than two gene expressions prior to the analysis, while the latter can only integrate the results obtained by applying the method to individual data sets.

In the following, based on the previous study (Taguchi, 2018a) where PCA-based unsupervised FE was employed, we try to integrate human and mouse midbrain development gene expression profiles to obtain key genes that contribute to this process, by applying TD-based unsupervised FE. It turned out that TD-based unsupervised FE can identify biologically more relevant and more common genes between human and mouse than can PCA-based unsupervised FE that outperformed other compared methods.

## Methods and Materials

### scRNA-seq Data

#### Midbrain Development of Humans and Mice

The first scRNA-seq data used in this study were downloaded from Gene Expression Omnibus (GEO) under the GEO ID GSE76381; the files named “GSE76381_EmbryoMoleculeCounts.cef.txt.gz” (for human) and “SE76381_MouseEmbryoMoleculeCounts.cef.txt.gz” (for mouse) were downloaded. These two gene expression profiles were generated from scRNA-seq data set: One represents human embryo ventral midbrain cells between 6 and 11 weeks of gestation (287 cells for 6 weeks, 131 cells for 7 weeks, 331 cells for 8 weeks, 322 cells for 9 weeks, 509 cells for 10 weeks, and 397 cells for 11 weeks, for a total of 1,977 cells). Another is a set of mouse ventral midbrain cells at six developmental stages between E11.5 and E18.5 (349 cells for E11.5, 350 cells for E12.5, 345 cells for E13.5, 308 cells for E14.5, 356 cells for E15.5, 142 cells for E18.5, and 57 cells for unknown, for a total of 1,907 cells).

#### Mouse Hypothalamus With and Without Acute Formalin Stress

The second scRNA-seq data used in this study were downloaded from GEO under GEOID GSE74672; the file named “GSE74672_expressed_mols_with_classes.xlsx.gz” was downloaded. It is generated from scRNA-seq data set that measures mouse hypothalamus with and without acute formalin stress. Various meta-data, which are included in the first 11 rows of the data set, are available. The meta-data available include sex, age, cell types [astrocytes, endothelial, ependymal, microglia, neurons, oligos, and vascular smooth muscle (VSM)], control vs stressed samples, and so on.

### TD-Based Unsupervised FE

#### Midbrain Development of Humans and Mice

TD-based unsupervised FE is a recently proposed method successfully applied to various biological problems. TD-based unsupervised FE can be used for integration of multiple measurements applied to the common set of genes. Suppose *x_ij_* ∈ ℝ*^N^*^×^*^M^* and *x_ik_* ∈ ℝ*^N^*^×^*^K^* are the *i*th expression of the *j*th and *k*th cells under the two distinct conditions (in the present study, they are human and mouse), respectively. Then the three-mode tensor, *x_ijk_* ∈ ℝ*^N^*^×^*^M^*^×^*^K^*, where *N* (= 13,889) is total number of common genes between human and mouse, which share gene symbols, *M* (= 1,977) is the number of human cells, and *K* (= 1,907) is total number of mouse cells, is defined as

(1) $$x_{ijk}=x_{ij}\cdot x_{ik}.$$

It is Case II Type I tensor (Taguchi, 2017e). Since it is too large to be decomposed, it is further transformed into Type II tensor, as follows:

(2) $$x_{jk}=\sum_{i=1}^{N}x_{ijk},$$

where $x_{jk}\in {\mathbb{R}}^{M\times K}$ is now not a tensor but a matrix. In this case, TD is equivalent to singular value decomposition (SVD). After applying SVD to *x_jk_* , we get SVD,

(3) $$x_{jk}=\overset{\min(M,\;K)}{\underset{\ell =1}{\sum }}\lambda_{\ell }u_{\ell j}v_{\ell k},$$

where $u_{\ell j}\in {\mathbb{R}}^{M\times M}$ and $v_{\ell k}\in {\mathbb{R}}^{K\times K}$ are singular value vectors attributed to cells of human scRNA-seq and those of mouse scRNA-seq, respectively. Here, Case II means that tensor is generated such that two matrices share the genes, while Type II means that summation is taken over as in Eq. (2). On the other hand, the tensor before taking summation as in Eq. (1) is Type I.

Singular value vectors attributed to genes of human and mouse scRNA-seq, $u_{\ell i}\in {\mathbb{R}}^{N\times M}$ and $v_{\ell i}\in {\mathbb{R}}^{N\times K}$ are defined as respectively.

(4) $$u_{\ell i}=\sum_{j=1}^{M}u_{\ell j}x_{ij},$$

(5) $$v_{\ell i}=\sum_{k=1}^{K}v_{\ell k}x_{ik},$$

In order to find genes associated with biological functions, we need to select *u_ℓj_* and *v_ℓk_* which are coincident with biological meaning. In this study, we employ time points of measurements as biological meanings. In other words, we seek for genes associated with time development. Since we would like to find any kind of time dependence, we simply deal with time points as un-ordered labeling. Thus, we apply categorical regression

(6) $$u_{\ell j}=a_{\ell }+\sum_{t=1}^{T}a_{\ell t}\delta_{jt},$$

(*T* = 6; *t* = 1 to *T*, which correspond to 6, 7, 8, 9, 10, and 11 weeks; see *Methods and Materials*) or

(7) $$v_{\ell k}=b_{\ell }+\sum_{t=1}^{T}b_{\ell t}\delta_{kt},$$

(*T* = 7; *t* = 1 to *T*, which correspond to E11.5, E12.5, E13.5, E14.5, F15.5, E18.5, and unknown; see *Methods and Materials*), where $\delta_{jt}\left(\delta_{kt}\right)=1$ when the *j*th (*k*th) cell is taken from the *t*th time point otherwise $\delta_{jt}\left(\delta_{kt}\right)=0$. *a_ℓ_*, *a_ℓt_*, *b_ℓ_* and *b_ℓt_* are the regression coefficients.

*P*-values are attributed to *ℓ*th singular value vectors using the above categorical regression [lm function in R (R Core Team, 2018) is used to compute *P*-values]. *P*-values attributed to singular value vectors are corrected by Benjamini-Hochberg (BH) criterion (Benjamini and Hochberg, 1995). Singular value vectors associated with corrected *P*-values of less than 0.01 are selected for the download analysis. Hereafter, the set of selected singular value vectors of human and mouse is denoted as $\Omega_{\ell }^{\text{human}}$ and $\Omega_{\ell }^{\text{mouse}}$, respectively.

*P*-values are attributed to genes with assuming χ^2^ distribution for the gene singular value vectors, *u_ℓi_* and *v_ℓi_*, corresponding to the cell singular value vectors selected by categorical regression as

(8) $$P_{i}^{\text{human}}=P_{\chi^{2}}\left[>\sum_{\ell \in {\text{Ω}}_{\ell }^{\text{human}}}{\left(\frac{u_{\ell i}-\langle u_{\ell i}\rangle }{\sigma_{\ell }^{\text{human}}}\right)}^{2}\right]$$

for human genes and

(9) $$P_{i}^{\text{mouse}}=P_{\chi^{2}}\left[>\sum_{\ell \in {\text{Ω}}_{\ell }^{\text{mouse}}}{\left(\frac{v_{\ell i}-\langle v_{\ell i}\rangle }{\sigma_{\ell }^{\text{mouse}}}\right)}^{2}\right]$$

for mouse genes, respectively. Here,

(10) $$\langle u_{\ell i}\rangle =\frac{1}{N}\sum_{i=1}^{N}u_{\ell i}$$

and

(11) $$\langle v_{\ell i}\rangle =\frac{1}{N}\sum_{i=1}^{N}v_{\ell i}.$$

$\sigma_{\ell }^{\text{human}}$ and $\sigma_{\ell }^{\text{mouse}}$ are the standard deviations of *ℓ*th gene singular value vectors for human and mouse, respectively, $\Omega_{\ell }^{\text{human}}$ and $\Omega_{\ell }^{\text{mouse}}$ are sets of *ℓ*s, selected by categorical regression for human [Eq. (6)] and mouse [Eq.(7)], respectively. $P_{\chi^{2}}[>x]$ is the cumulative probability of χ^2^ distribution when the argument takes values larger than *x*. $P_{i}^{\text{human}}$ and $P_{i}^{\text{mouse}}$ are corrected by BH criterion, and genes associated with corrected *P*-values of less than 0.01 are selected.

#### Mouse Hypothalamus With and Without Acute Formalin Stress

The application of TD-based unsupervised FE to mouse hypothalamus is quite similar to that of mouse and human midbrain. There are also two matrices, $x_{ij}\in {\mathbb{R}}^{N\times M}$ and $x_{ik}\in {\mathbb{R}}^{N\times K}$ which correspond to the *i*th expression of the *j*th and *k*th cells under the two distinct conditions (in the present case, they are without and with acute formalin stress, respectively); *N*=24,341,*M*=1,785 and *K*=1,096. Case II Type II tensor, *x_jk_*, was also generated using Eqs. (1) and (2), and SVD was applied to *x_jk_* as Eq. (3). Then singular value vectors attributed to genes of samples without and with acute formalin stress, *u_ℓi_* and *v_ℓi_*, were computed by Eqs. (4) and (5). We also applied categorical regressions to *u_ℓi_*. and *v_ℓi_*, although categories considered here are not time points but cell types. Then categorical regressions applied to *u_ℓi_* and *v_ℓi_* in mouse hypothalamus without and with acute formalin stress are

(12) $$u_{\ell j}=a_{\ell }+\overset{7}{\underset{s=1}{\sum }}a_{\ell s}\delta_{js},$$

(13) $$v_{\ell k}=b_{\ell }+\overset{7}{\underset{s=1}{\sum }}b_{\ell s}\delta_{ks},$$

where *s* stands for one of seven cell types mentioned in Methods and Materials and $\delta_{js}\left(\delta_{ks}\right)=1$ when the *j*th (*k*th) cell is taken from the *s*th cell types otherwise $\delta_{js}\left(\delta_{ks}\right)=0$. **Table 1** lists the number of cells in these categories. The remaining procedures to select genes associated with identified cell type dependency are exactly the same as those in midbrain case.

**Table 1:** The number of cells that belong to either without or with acute formalin stress or cell types.

Cell types	Without	With
	Acute stress	
Astrocytes	135	132
Endothelial	169	71
Ependymal	211	145
Microglia	34	14
Neurons	628	270
Oligos	570	431
VSM	38	33

VSM, vascular smooth muscle.

### Enrichment Analyses

Various enrichment analysis methods are performed with separate uploading selected human and mouse gene symbols, or genes selected commonly between samples without and samples with acute formalin stress, to Enrichr (Kuleshov et al., 2016).

## Results

### Midbrain Development of Humans and Mice

As a result, following the procedure described in the *Methods and Materials*, we identified 55 and 44 singular value vectors attributed to cells, *u_ℓj_*s and *v_ℓk_*s for human and mouse, respectively. One possible validation of selected *u_ℓj_*s and *v_ℓk_*s is coincidence. Although cells measured are not related between human and mouse at all, if SVD works well, corresponding singular value vectors (i.e., *u_ℓj_* and *v_ℓk_* sharing the same *ℓ*s) attributed to cells should share something biological, for example, time dependence. This suggests that it is more likely that corresponding singular value vectors attributed to cells, *u_ℓj_* and *v_ℓk_*, are simultaneously associated with significant *P*-values computed by categorical regression. As expected, they are highly significantly correlated. **Table 2** shows confusion matrix of the coincidence of selected singular value vectors between human and mouse. For human cells, only the top 1,907 singular value vectors among all 1,977 singular value vectors are considered, since the total number of singular value vectors attributed to mouse cells is 1,907.

**Table 2:** Confusion matrix of coincidence between selected 55 singular value vectors selected among all 1,977 singular value vectors, *u_ℓj_*, attributed to human cells and 44 singular value vectors selected among all 1907 singular value vectors, *v_ℓk_*, attributed to mouse cells.

		Human	
		Not selected	Selected
Mouse	Not selected	1,833	12
	Selected	23	32

Selected: corrected P-values, computed with regression analysis [Eqs. (6) and (7)], are less than 0.01. Not selected: otherwise. Odds ratio is as many as 227, and P-values computed by Fisher’s exact test are 1.44×10^-44^.

**Figure 1:**
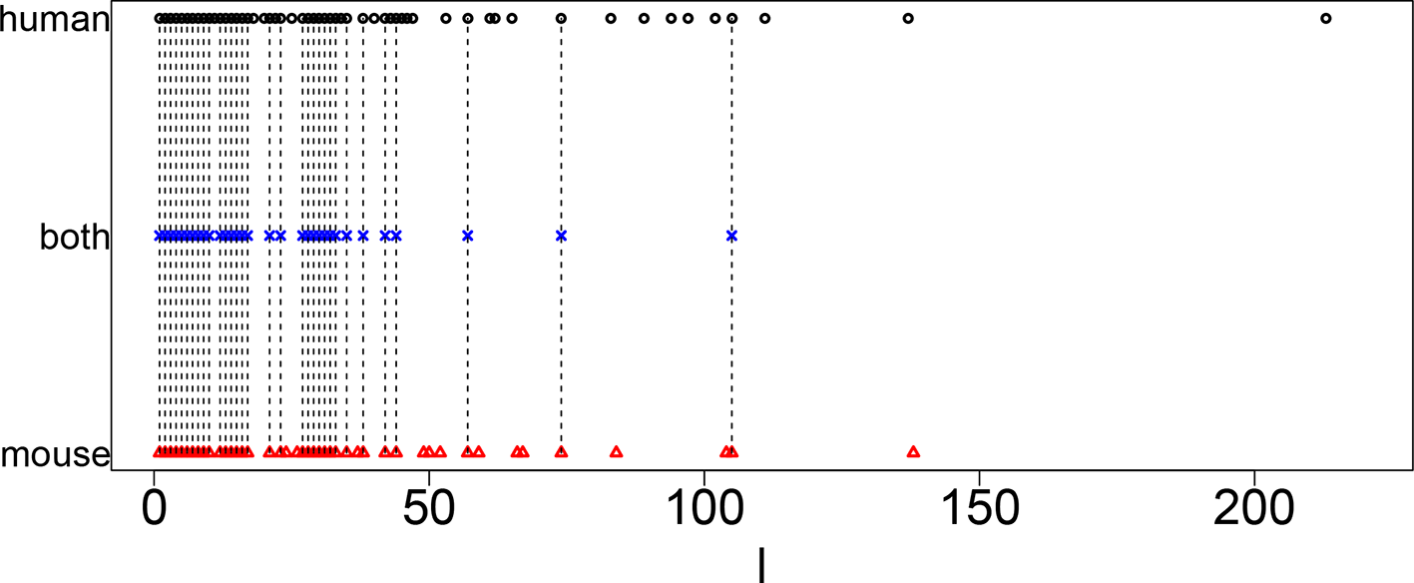
Coincidence between singular value vectors shown in **Table 2**. Horizontal axis: singular value vector numbering *ℓ*. Black open circles, *ℓ* selected for human; blue crosses, *ℓ* selected for both human and mouse; red open triangles, *ℓ* selected for mouse. Vertical black broken lines connect *ℓ* selected for both human and mouse.

**Figure 1** shows the coincidence of selected singular value vectors between human and mouse. Singular value vectors with smaller *ℓ*s, that is, with more contributions, are more likely selected and coincident between human and mouse. This can be the side evidence that guarantees that TD-based unsupervised FE successfully integrated human and mouse scRNA-seq data.

Next, we selected genes with following the procedures described in *Methods and Materials*. (The list of genes is available as **Supplementary Data Sheet 1** and **2**). The first validation of selected genes is the coincidence between human and mouse. In Taguchi’s previous study (Taguchi, 2018a), more number of common genes were selected by PCA-based unsupervised FE than other methods compared, that is, highly variables genes, bimodal genes, and dpFeature. **Table 3** shows the confusion matrix that describes the coincidence of selected genes between human and mouse. Odds ratio is as large as 133, and *P*-value is 0 (i.e., less than numerical accuracy), which is significantly better than coincidence of selected genes between human and mouse (53 common genes between 116 genes selected for human and 118 genes selected mouse), previously achieved by PCA-based unsupervised FE (Taguchi, 2018a), which outperformed other methods, that is, highly variable genes, bimodal genes, and dpFeature.

**Table 3:** Confusion matrix of coincidence between selected 456 genes for human and selected 505 genes for mouse among all 13,384 common genes.

		Human	
		Not selected	Selected
Mouse	Not selected	13,233	151
	Selected	200	305

Selected: corrected P-values, computed with χ^2^ distribution [Eqs. (8) and (9)], are less than 0.01. Not selected: otherwise. Odds ratio is as many as 133, and P-values computed by Fisher’s exact test are 0 (i.e., less than numerical accuracy).

On the other hand, most of the genes selected by PCA-based unsupervised FE in the previous study (Taguchi, 2018a) are included in the genes selected by TD-based unsupervised FE in the present study. One hundred two genes are selected by TD-based unsupervised FE among 116 human genes selected by PCA-based unsupervised FE in the previous study (Taguchi, 2018a), while 91 genes are selected by TD-based unsupervised FE among 118 mouse genes by PCA-based unsupervised FE. Thus, TD-based unsupervised FE is quite consistent with PCA-based unsupervised FE.

Biological significance tested by enrichment analysis is further enhanced (Full list of enrichment analysis is available as **Supplementary Tables 1** and **2**). Most remarkable advance achieved by TD-based unsupervised FE is “Allen Brain Atlas,” to which only downregulated genes were enriched in the previous study (Taguchi, 2018a). As can be seen in **Table 4**, now much enrichment is associated with upregulated genes. In addition to this, most of the five top-ranked terms are related to paraventricular nucleus, which is adjusted to midbrain. This suggests that TD-based unsupervised FE successfully identified genes related to midbrain.

**Table 4:** Five top-ranked terms from “Allen Brain Atlas up” by Enrichr for selected 456 human genes and 505 mouse genes.

Human			
Term	Overlap	*P*-value	Adjusted *P* -value
Paraventricular hypothalamic nucleus, magnocellular division, medial magnocellular part	31/301	2.68 × 10^–12^	2.91 × 10^–9^
Paraventricular hypothalamic nucleus, magnocellular division	31/301	2.68 × 10^-12^	2.91 × 10^-9^
Paraventricular hypothalamic nucleus, magnocellular division, posterior magnocellular part	28/301	3.39 × 10^-10^	1.47 × 10^-7^
Paraventricular hypothalamic nucleus	29/301	7.02 × 10^-11^	5.08 × 10^-8^
Paraventricular nucleus, dorsal part	27/301	1.57 × 10^-9^	4.88 × 10^-7^
Mouse			
Paraventricular hypothalamic nucleus, magnocellular division, medial magnocellular part	31/301	4.03 × 10^-11^	2.19 × 10^-8^
Paraventricular hypothalamic nucleus, magnocellular division	31/301	4.03 × 10^-11^	2.19 × 10^-8^
Paraventricular hypothalamic nucleus, magnocellular division, posterior magnocellular part	31/301	4.03 × 10^-11^	2.19 × 10^-8^
Lower dorsal lateral hypothalamic area	29/301	8.40 × 10^-10^	3.65 × 10^-7^
Paraventricular hypothalamic nucleus, magnocellular division, posterior magnocellular part, lateral zone	31/301	4.03 × 10^-11^	2.19 × 10^-8^

In addition to this, “Jensen TISSUES” (**Table 5**) for Embryonic_brain is highly enhanced [i.e., more significant (smaller), with *P*-values ∼10^-100^ which were as large as 10^-10^ to 10^-20^ in the previous study (Taguchi, 2018a)]. On the other hand, “ARCHS4 tissues” also strongly supports the biological reliability of selected genes (**Table 6**). The term “MIDBRAIN” is enriched highly, and it is top ranked for both human and mouse.

**Table 5:** Enrichment of embryonic brain by “JENSEN TISSUES” in Enrichr.

Term	Overlap	*P* -value	Adjusted *P*-value
Human			
Embryonic_brain	330/4936	3.36 × 10^-104^	4.30 × 10^-102^
Mouse			
Embryonic_brain	366/4936	3.59 × 10^-115^	4.59 × 10^-113^

**Table 6:** Enrichment of embryonic brain by “ARCHS4 Tissues” in Enrichr.

Term	Overlap	*P* -value	Adjusted *P*-value
Human			
MIDBRAIN	248/2316	1.02 × 10^-129^	1.11 × 10^-127^
Mouse			
MIDBRAIN	248/2316	1.44 × 10^-99^	1.56 × 10^-97^

There is some brain-related enrichment found in other categories, although it is not strong enough compared with that of the top three. Brain-related terms in “GTEx Tissue Sample Gene Expression Profiles up” (**Table 7**) are also enhanced for mouse brain (top three terms are brain), although no brain terms are enriched within five top-ranked terms for human (this discrepancy cannot be understood at the moment). On the contrary, brain-related terms in “MGI Mammalian Phenotype 2017” (**Table 8**) are enhanced for human brain (fourth and fifth ranks), although no brain terms are enriched within the five top-ranked terms for mouse (this discrepancy also cannot be understood at the moment). The above observations suggest that TD-based unsupervised FE could identify genes related to mouse and human embryonic midbrain.

**Table 7:** Five top-ranked terms from “GTEx Tissue Sample Gene Expression Profiles up” by Enrichr for selected 456 human genes and 505 mouse genes. Brain-related terms are asterisked.

Human			
Term	Overlap	*P* -value	Adjusted *P* -value
GTEX-QCQG-1426-SM-48U22_ovary_female_50-59_years	105/1165	3.56 × 10^-35^	1.04 × 10^-31^
GTEX-RWS6-1026-SM-47JXD_ovary_female_60-69_years	116/1574	7.96 × 10^-31^	7.74 × 10^-28^
GTEX-TMMY-1726-SM-4DXTD_ovary_female_40-49_years	117/1582	2.97 × 10^-31^	4.33 × 10^-28^
GTEX-RU72-0008-SM-46MV8_skin_female_50-59_years	94/1103	1.99 × 10^-31^	1.45 × 10^-26^
GTEX-R55E-0008-SM-48FCG_skin_male_20-29_years	111/1599	3.67 × 10^-27^	1.78 × 10^-24^
*GX-WVLH-0011-R4A-SM-3MJFS_brain_male_50-59_years	139/1957	1.93 × 10^-30^	5.63 × 10^-27^
*GX-X261-0011-R8A-SM-4E3I5_brain_male_50-59_years	135/1878	5.24 × 10^-30^	7.65 × 10^-27^
*GX-T5JC-0011-R4A-SM-32PLT_brain_male_20-29_years	129/1948	3.51 × 10^-25^	3.42 × 10^-22^
Mouse			
GTEX-R55E-0008-SM-48FCG_skin_male_20-29_years	109/1599	4.93 × 10^-22^	2.40 × 10^-19^
GTEX-TMMY-1726-SM-4DXTD_ovary_female_40-49_years	107/1582	2.37 × 10^-21^	7.69 × 10^-19^

**Table 8:** Five top-ranked terms from “MGI Mammalian Phenotype 2017” by Enrichr for selected 456 human genes and 505 mouse genes. Brain-related terms are asterisked.

Human			
Term	Overlap	*P* -value	Adjusted *P* -value
MP:0002169_no_abnormal_phenotype_detected	82/1674	2.52 × 10^-11^	5.53 × 10^-8^
MP:0001262_decreased_body_weight	63/1189	3.40 × 10^-10^	3.72 × 10^-7^
MP:0001265_decreased_body_size	46/774	3.20 × 10^-9^	2.33 × *s*10^-6^
*M0009937_abnormal_neuron_differentiation	15/106	1.81 × 10^-8^	9.90 × 10^-6^
*M0000788_abnormal_cerebral_cortex_morphology	17/145	3.64 × 10^-8^	1.60 × 10^-5^
Mouse			
MP:0002169_no_abnormal_phenotype_detected	89/1674	1.36 × *s*10^-11^	3.09 × 10^-8^
MP:0011091_prenatal_lethality,_complete_penetrance	27/272	1.68 × 10^-9^	1.91 × 10^-6^
MP:0001262_decreased_body_weight	65/1189	3.93 × 10^-9^	2.97 × 10^-6^
MP:0011100_preweaning_lethality,_complete_penetrance	42/674	8.55 × 10^-8^	3.88 × 10^-5^
MP:0001265_decreased_body_size	46/774	8.22 × 10^-8^	3.88 × 10^-5^

We also uploaded selected 456 human genes and 505 mouse genes to STRING server (Szklarczyk et al., 2014), which evaluates protein–protein interaction (PPI) enrichment. Among 456 human genes, 7,488 PPI are reported, while the expected number of PPI is as small as 3,524 (*P*-value is less than 1×10^-6^). Among 505 mouse genes, 6,788 PPI are reported, while the expected number of PPI is as small as 3,290 (*P*-value is again less than 1×10^-6^). Thus, TD-based unsupervised FE can successfully identify significantly interacting protein-coding genes.

**Figure 2:**
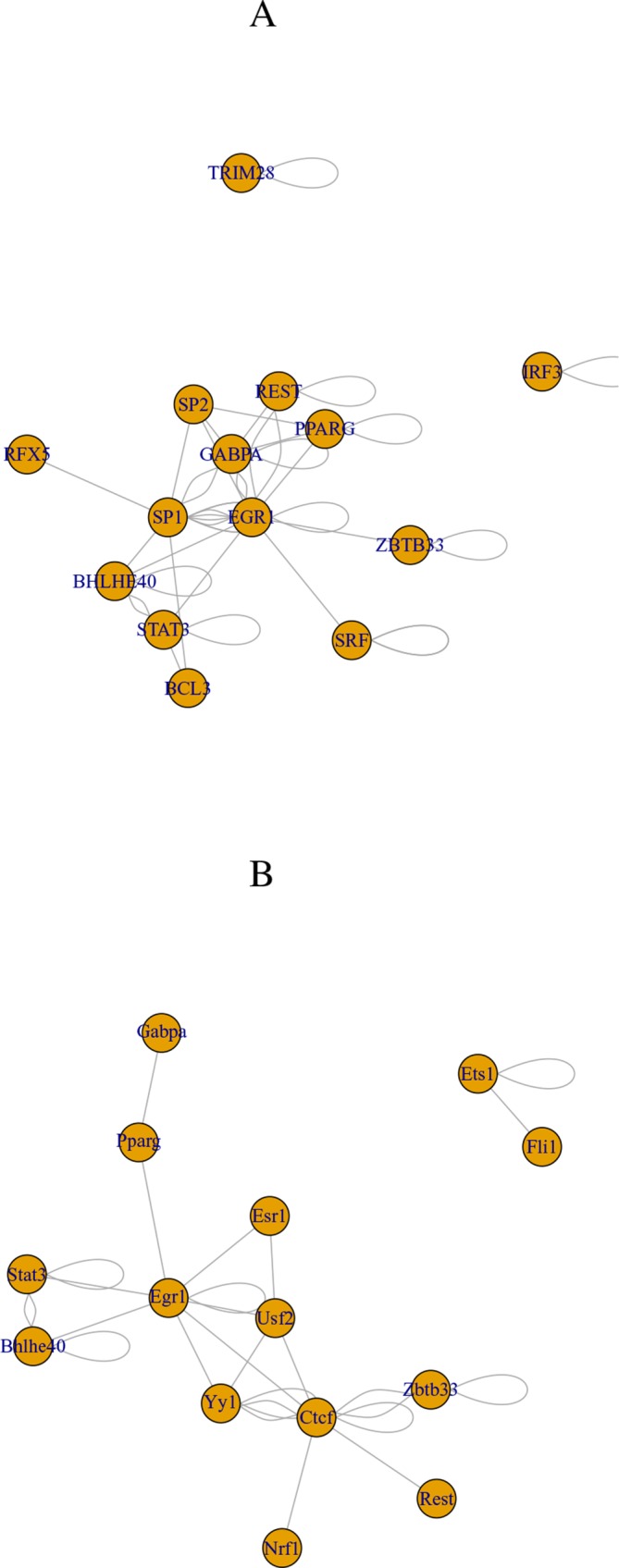
Transcription factor (TF) network identified by regnetworkweb for TFs in **Table 9**. **(A)** Human and **(B)** mouse.

Finally, we checked if transcription factors (TFs) that target selected genes are common between human and mouse (**Table 9**). These TFs are associated with adjusted *P*-values of less than 0.01 in “ENCODE and ChEA Consensus TFs from ChIP-X” of Enrichr. They are highly overlapped between human and mouse (there are 10 common TFs between 16 TFs found in human and 24 TFs found in mouse). Although selected TFs are very distinct from those in the previous study (Taguchi, 2018a), they are highly interrelated with each other (see below). These TFs are uploaded to the regnetworkweb server (Liu et al., 2015), and TF networks shown in **Figure 2** are identified. Clearly, even partially, these TFs interact highly with each other.

**Table 9:** TFs enriched in “ENCODE and ChEA Consensus TFs from ChIP-X” by Enrichr for human and mouse. Bold TFs are common.

Human	BCL3, BHLHE40, EGR1, GABPA, IRF3, PPARG, REST, RFX5, SP1, SP2, SRF, STAT3, TCF7L2, TRIM28, TRIM28, ZBTB33
Mouse	BHLHE40, CTCF, E2F4, E2F6, EGR1, ESR1, ETS1, FLI1, GABPA, IRF3, NFIC, NRF1, PPARG, RCOR1, REST, RFX5, SPI1, STAT3, TCF7L2, USF1, USF2, YY1, ZBTB33, ZNF384


TFs, transcription factors.

We also checked if the 10 commonly selected TFs (in bold in **Table 9**) are related to brains. Lack of BHLHE40 was found to result in brain malfunction (Hamilton et al., 2018). The function of EGR1 was found in embryonic rat brain (Wells et al., 2011). GABPA is essential for human cognition (Reiff et al., 2014). IRF3 is related to brain disease (Schultz et al., 2019). PPAR, which PPARG belongs to, is believed to be the therapeutic target of neurodegenerative diseases (Warden et al., 2016). REST is a master regulator of neurogenesis (Mozzi et al., 2017). RFX5 is known to be expressive in fetal brain (Sugiaman-Trapman et al., 2018). STAT3 promotes brain metastasis (Priego et al., 2018). TCF7L2 regulates brain gene expression (Shao et al., 2013). ZBTB33 affects the mouse behavior through regulating brain gene expression (Kulikov et al., 2016). Thus, all 10 commonly selected TFs are related to brains.

### Mouse Hypothalamus With and Without Acute Formalin Stress

Although the effectiveness of the proposed strategy toward scRNA-seq is obvious in the results shown in the previous subsection, one might wonder if it is accidental. In order to dispel such doubts, we apply TD-based unsupervised FE to yet another scRNA-seq data set: mouse hypothalamus with and without acute formalin stress. Contrary to the data set analyzed in the previous subsection where very distant two data sets were analyzed, the data sets analyzed here are very close to each other. Both data sets are taken from the same tissue of mouse, hypothalamus. The only difference is if they are stressed by formalin dope or not. The motivation why we here specifically apply TD-based unsupervised FE to two close data sets is as follows: When two data sets are too close, it might be difficult to identify which genes are commonly altered by additional condition, in this case, the dependence upon cell types, because all genes might behave equally between the two. Thus, it is not a bad idea to check if TD-based unsupervised FE can work well when not only very distant data sets are analyzed but also very close data sets are analyzed.

With following the procedure described in the Materials and Methods, we identified 30 and 24 singular value vectors attributed to cells, *u_ℓj_*s and *v_ℓk_*s, without and with acute formalin stress, respectively. We again applied Fisher’s exact test (**Table 10**). Although odds ratio is 10 times larger than that in **Table 2**, *P* -value is even smaller than that in **Table 2**; this suggests that TD-based unsupervised FE could identify not all of genes but only limited genes as being common between two experimental conditions: without and with stress.

**Table 10:** Confusion matrix of coincidence between selected 30 singular value vectors selected among all 1,096 singular value vectors, *u_ℓj_* ,attributed to samples without stress and 24 singular value vectors selected among all 1,096 singular value vectors, *v_ℓk_* attributed to samples with stress.

		Not selected	Selected
Without stress	Not selected	1,065	1
	Selected	7	23

For samples without stress, only the top 1,096 singular value vectors among all 1,785 singular value vectors are considered, since total number of singular value vectors attributed to samples without stress is 1,096. Selected: corrected P -values, computed with regression analysis (Eqs. (12) and (13)), are less than 0.01. Not selected: otherwise. Odds ratio is as many as 2,483, and P-values computed by Fisher’s exact test are 1.92×10^-40^.

**Figure 3:**
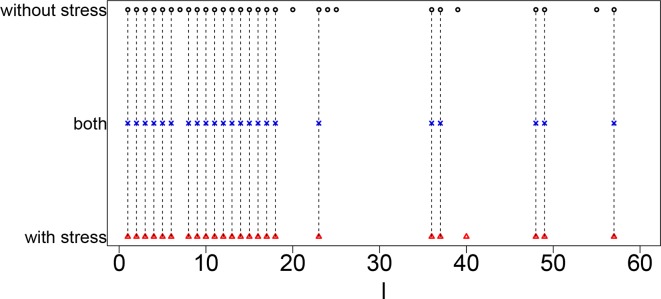
Coincidence between singular value vectors shown in **Table 10**. Horizontal axis: singular value vector numbering *ℓ*. Black open circles, *ℓ*s selected for samples without stress; blue crosses, *ℓ*s selected for both samples without and with stress; red open triangles, *ℓ*s selected for samples with stress. Vertical black broken lines connect *ℓ*s selected for both samples without and with stress.

**Figure 3** shows the coincidence of selected singular value vectors between samples without and with stress. Singular value vectors with smaller *ℓ*s, that is, with more contributions, are more likely selected and coincident between samples without and with stress. This can be the side evidence that guarantees that TD-based unsupervised FE successfully integrated scRNA-seq data taken from samples without and with stress while avoiding to regard that all are coincident between two samples.

Next, we selected genes with following the procedures described in the *Methods and Materials*. The first validation of selected genes is the coincidence between human and mouse. **Table 11** shows the confusion matrix that describes the coincidence of selected genes between samples without and with stress. Odds ratio is as large as 270, and *P*-value is 0 (i.e., less than numerical accuracy). Thus, as expected, TD-based unsupervised FE could not identify all genes but only a limited number of genes associated with cell-type dependence.

**Table 11:** Confusion matrix of coincidence between selected 4,150 genes for samples without stress and selected 3,621 genes for samples with stress among all 24,341 genes.

		With stress	
		Not selected	Selected
Without stress	Not selected	19,894	297
	Selected	826	3,324

Selected: corrected P -values, computed with χ^2^s atribution that corresponds to Eqs. (8) and (9) in human and mouse midbrain study, are less than 0.01. Not selected: otherwise. Odds ratio is as many as 270, and P-values computed by Fisher’s exact test are 0 (i.e., less than numerical accuracy).

Finally, we tried to evaluate if genes selected are tissue type specific, that is, hypothalamus. We have uploaded 3,324 commonly selected genes to Enrichr. “GTEx Tissue Sample Gene Expression Profiles up” suggest that all five top-ranked terms are brain with high significance (**Table 12**, adjusted *P*-values are less than 1×10^-130^). This suggests that TD-based unsupervised FE successfully identified limited number of genes related to brains even using closely related samples. In order to be more specific, we checked “Allen Brain Atlas up” in Enrichr. Then we found that all five top-ranked terms are hypothalamic (**Table 13**). It is interesting that TD-based unsupervised FE could successfully identify hypothalamus-specific genes by only using scRNA-seq retrieved from hypothalamus. It is usually required to use data taken from other tissues in order to identify tissue-specific genes because we need to compare targeted tissues and not targeted tissues in order to identify genes expressed specifically in target tissues. The successful identification of genes specific to something without using the comparison with other samples was also observed previously during an attempt to identify tumor-specific genes by TD-based unsupervised FE (Taguchi, 2017c). In this sense, TD-based unsupervised FE methods are effective not only when genes common between two distinct conditions are sought but also when genes common between two closely related conditions are sought. Thus, it is unlikely that the success of a TD-based unsupervised method applied to scRNA-seq is accidental.

**Table 12:** Five top-ranked terms from “GTEx Tissue Sample Gene Expression Profiles up” by Enrichr for 3,324 genes selected commonly between samples without and with stress.

Term	Overlap	*P* -value	Adjusted *P* -value
GTEX-WWYW-0011-R10A-SM-3NB35_brain_female_50-59_years	1006/2885	2.7880 × 10^-151^	8.135 × 10^-148^
GTEX-T6MN-0011-R1A-SM-32QOY_brain_male_50-59_years	859/2317	2.9865 × 10^-144^	4.3575 × 10^-141^
GTEX-QVUS-0011-R3A-SM-3GAFD_brain_female_60-69_years	963/2759	6.8195 × 10^-144^	6.6325 × 10^-141^
GTEX-T2IS-0011-R3A-SM-32QPB_brain_female_20-29_years	967/2792	5.5265 × 10^-142^	4.0315 × 10^-139^
GTEX-WZTO-0011-R3B-SM-3NMC6_brain_male_40-49_years	991/2972	2.6805 × 10^-133^	1.5645 × 10^-130^

**Table 13:** Five top-ranked terms from “Allen Brain Atlas up” by Enrichr for 3,324 genes selected commonly between samples without and with stress.

Term	Overlap	*P* -value	Adjusted *P* -value
Paraventricular hypothalamic nucleus	120/301	3.38 × 10-^22^	7.41 × 10^-19^
Paraventricular hypothalamic nucleus, parvicellular division	119/301	1.15 × 10^-21^	1.27 × 10^-18^
Paraventricular hypothalamic nucleus, parvicellular division, medial parvicellular part, dorsal zone	117/301	1.29 × 10^-20^	9.42 × 10^-18^
Paraventricular nucleus, cap part	116/301	4.22 × 10^-20^	2.31 × 10^-17^
Paraventricular hypothalamic nucleus, magnocellular division	115/301	1.36 × 10^-19^	5.96 × 10^-17^

## Discussions and Future Work

In this study, we applied TD-based unsupervised FE to the integration of scRNA-seq data sets taken from two species: human and mouse. In the sense of identification of biologically more relevant set of genes, TD-based unsupervised FE can outperform PCA-based unsupervised FE that previously (Taguchi, 2018a) could outperform three more popular methods: highly variable genes, bimodal genes, and dpFeature. Thus, it is expected that TD-based unsupervised FE can do so, too.

For the purpose of integration of two scRNA-seq data sets, TD-based unsupervised FE has many advantages than the other four methods, that is, PCA-based unsupervised FE, highly variable genes, bimodal genes, and dpFeature. At first, TD-based unsupervised FE can integrate two scRNA-seq data sets, not after but before the selection of genes. This enabled us to identify more coincident gene sets between two scRNA-seq in this study of human and mouse. As a result, we were able to identify more coincident results between human and mouse.

The criteria of gene selection are quite robust; they should be dependent upon time points when they are measured. We did not have to specify how they are actually correlated with time. It is another advantage of TD-based unsupervised FE.

By applying enrichment analysis to the genes selected, we found many valuable insights about the biological process. As a result, we identified 10 key TFs that might regulate embryonic midbrain developments. All of the 10 selected TFs turned out to be related to brains.

TD-based unsupervised FE turned out to be quite effective to integrate two scRNA-seq data sets. This method should be applied to various scRNA-seq data sets considering broader scope of investigations.

In future work, we plan to (1) utilize the proposed TD-based unsupervised FE under the transfer learning setting; (2) extend the proposed approach to handle the data integration from multiple related tasks; and (3) investigate the performance of the proposed approach when coupled with machine and deep learning algorithms.

## Data Availability

The data sets analyzed for this study can be found in the GEO.

https://www.ncbi.nlm.nih.gov/geo/query/acc.cgi?acc=GSE76381.

https://www.ncbi.nlm.nih.gov/geo/query/acc.cgi?acc=GSE74672

## Author Contributions

Y-HT planned the research, performed analyses, and wrote a paper. TT discussed the results and wrote a paper.

## Funding

This study was supported by KAKENHI (17K00417 and 19H05270) and Okawa Foundation (grant number 17-10). This project was also funded by the Deanship of Scientific Research (DSR) at King Abdulaziz University, Jeddah, under grant no. KEP-8-611-38. The authors, therefore, acknowledge with thanks DSR for technical and financial support.

## Conflict of Interest Statement

The authors declare that the research was conducted in the absence of any commercial or financial relationships that could be construed as a potential conflict of interest.
